# Evidence from facility level inputs to improve quality of care for maternal and newborn health: interventions and findings

**DOI:** 10.1186/1742-4755-11-S2-S4

**Published:** 2014-09-04

**Authors:** Jai K Das, Rohail Kumar, Rehana A Salam, Zohra S Lassi, Zulfiqar A Bhutta

**Affiliations:** 1 Division of Women & Child Health, Aga Khan University, Karachi, Pakistan; 2 Program for Global Pediatric Research, Hospital For Sick Children, Toronto

**Keywords:** Facility, maternal, newborn, quality of care, obstetric care, delivery, social support, staffing

## Abstract

Most of the maternal and newborn deaths occur at birth or within 24 hours of birth. Therefore, essential lifesaving interventions need to be delivered at basic or comprehensive emergency obstetric care facilities. Facilities provide complex interventions including advice on referrals, post discharge care, long-term management of chronic conditions along with staff training, managerial and administrative support to other facilities. This paper reviews the effectiveness of facility level inputs for improving maternal and newborn health outcomes. We considered all available systematic reviews published before May 2013 on the pre-defined facility level interventions and included 32 systematic reviews.

Findings suggest that additional social support during pregnancy and labour significantly decreased the risk of antenatal hospital admission, intrapartum analgesia, dissatisfaction, labour duration, cesarean delivery and instrumental vaginal birth. However, it did not have any impact on pregnancy outcomes. Continued midwifery care from early pregnancy to postpartum period was associated with reduced medical procedures during labour and shorter length of stay. Facility based stress training and management interventions to maintain well performing and motivated workforce, significantly reduced job stress and improved job satisfaction while the interventions tailored to address identified barriers to change improved the desired practice. We found limited and inconclusive evidence for the impacts of physical environment, exit interviews and organizational culture modifications.

At the facility level, specialized midwifery teams and social support during pregnancy and labour have demonstrated conclusive benefits in improving maternal newborn health outcomes. However, the generalizability of these findings is limited to high income countries. Future programs in resource limited settings should utilize these findings to implement relevant interventions tailored to their needs.

## Background

Most of the maternal and newborn deaths occur at birth or within 24 hours of birth; therefore essential lifesaving interventions need to be delivered at basic or comprehensive emergency obstetric and newborn care (BEmONC /CEmONC) facilities [1-4]. Facilities provide critical emergency care during labor and delivery, hence strengthening health facilities and referral linkages between communities and facilities is vital. Facilities should be equipped with commodities and skilled personnel to provide minimum required standard care for women and newborns in need of obstetric and special care. They should be able to provide the defined minimal 'signal functions' that are the key interventions for treating vast majority of maternal complications and for resuscitation of the newborn after birth (Table 1) [5]. The list of signal functions is not exhaustive but these functions serve as indicators of the level of care being provided. It is estimated that providing these essential interventions at scale (over 90% coverage) in communities and facilities can reduce the neonatal mortality rate by 70% [6,7]. Although facility-based care during childbirth typically requires more resources than home-based care, it is often more cost-effective in preventing deaths [8].

**Table 1:** Signal functions used to identify basic and comprehensive emergency obstetric care services

Basic services	Comprehensive services
(1) Administer parenteral antibiotics	Perform signal functions 1–7, plus:

(2) Administer uterotonic drugs (i.e. parenteral oxytocin)	(8) Perform surgery (e.g. caesarean section)

(3) Administer parenteral anticonvulsants for preeclampsia and eclampsia (i.e. magnesium sulfate).	(9) Perform blood transfusion

(4) Manually remove the placenta	

(5) Remove retained products (e.g. manual vacuum extraction, dilation and curettage)	

(6) Perform assisted vaginal delivery (e.g. vacuum extraction, forceps delivery)	

(7) Perform basic neonatal resuscitation (e.g. with bag and mask)	

A basic emergency obstetric care facility is one in which all functions 1–7 are performed. A comprehensive emergency obstetric care facility is one in which all functions 1–9 are performed.	

Alongside emergency obstetric care; facilities provide complex clinical care interventions including referrals, post discharge care, long-term management of chronic conditions and managerial and administrative support to other facilities. They also serve as gateways for drugs and medical supplies, laboratory testing services, general procurement and data collection from health information systems. Facilities also disseminate technologies by training new staff and providing continuing professional education for existing staff at different facilities. In this review, we aim to systematically review and summarize the available evidence from relevant systematic reviews on the impacts of the outlined facility level inputs (Table 2) to improve the quality of care for maternal and newborn health (MNH). For this review we have broadly categorized these interventions into four categories: interventions for well performing and motivated work force; interpersonal care and social support; safety culture; and staffing models.

**Table 2:** Components of facility level interventions

**Well performing and motivated workforce:** includes various strategies to manage and cope with job stress, managing dual practice among healthcare workers, exit interview and any structural or cultural modification in the healthcare environment. **Interpersonal care and social support:** These are interventions provided by professionals or non-professionals aimed at improving psychological well-being of patients as well as healthcare workers. Pregnancy, perinatal deaths, childbirth and parenting are some of the specific phenomena that require continuous social support. **Safety culture:** Facility based safety culture includes any intervention to enhance the safety of healthcare workers and patients in healthcare environment including hand hygiene promotion, interventions to reduce medication errors and preventive vaccinations for the health care professionals. **Staffing models:** These are organizational interventions for staff management including skill, qualification or grade mix, maintaining staff-patient ratios and measures for improving collaboration between two or more health and/or social care professionals.

## Facility level characteristics

### Well performing and motivated workforce

The quality of health service delivery depends on the willingness and drive of health workers to perform their tasks, adequate resources, and health workers’ competency [9]. Interventions to maintain workforce motivation and enhance performance include support to manage and deal with job stressors, policies for dual practice among healthcare workers, conducting exit interviews, and modifications in the organizational infrastructure and work environment to improve healthcare worker performance. These interventions provide support at the individual level as well as the interface between the health worker and the organization [10]. Several studies have emphasized the importance of policy and procedural changes to improve performance and promote evidence based practice [11-14]. Organizational culture also plays a major role in maintaining motivated workforce and encompasses multiple aspects of beliefs, values, norms of behavior, routines and traditions [15]. Organizational culture alongside structural reforms have been suggested to achieve effective improvement in healthcare performance [16,17] however, it is not as straight forward owing to the reluctance to change. Hence, it is important to identify and overcome the barriers to change prior to the implementation. The effects of attempts to translate research evidence into practice and improve performance for these interventions remain inconsistent [18,19].

### Interpersonal care and social support

Interventions to enhance interpersonal care and social support include interventions provided by professionals or non-professionals aimed at improving psychological well-being of patients as well as healthcare workers. Pregnancy, perinatal deaths, childbirth and parenting are some of the specific phenomenon that requires continuous social support. Common elements of this care include emotional support, information about labour progress and advice regarding coping techniques, comfort measures and advocacy. It is reported to contribute substantially to women’s satisfaction with the childbirth experience and provides both direct and buffering effects in decreasing stress and promoting health and coping [20-23].

### Safety culture

Developing a culture of safety is a core element of many efforts to improve patient safety and care quality in emergency care settings. Recently, there has been a major focus on measuring and improving safety culture to enhance patient and provider safety in healthcare facilities [24]. It involves any intervention to enhance safety in healthcare environment including hand hygiene promotion, interventions to reduce medication errors and preventive vaccination (like influenza) administered to health care professionals. Several studies show that safety culture and the related concept of safety climate are associated with improved error reporting, reductions in adverse events, and mortality [24-26]. Despite being widely implemented, there has been limited evidence of the effectiveness of these interventions within hospitals, hence it is important to determine the extent to which they are effective, generalizable and sustainable for rational allocation of resources [27].

### Staffing models

These are organizational interventions for staff management including skills, qualification or grade mix, maintaining staff-patient ratios, and measures for improving collaboration between two or more health and/or social care professionals. An emerging challenge in this domain is determining the most effective mix of staff and skills needed to deliver quality and cost-effective patient care in the light of rising demand for health services, cost containment, and staff shortages [28-31].

## Methods

We considered all available systematic reviews on the pre-defined facility level interventions published before May 2013 as outlined in our conceptual framework [32]. A separate search strategy was developed for each component using pre-identified broad keywords, medical subject heading (MeSH) and free text terms:[(Performance OR motivation OR support OR “social support” OR “interpersonal care” OR labour OR labor OR safety OR “safety culture” OR “environmental safety” “health professional” OR “health care worker” OR “healthcare professional*” OR “staffing models” OR “staffing ratios” OR “nurse-patient” OR staff* OR “skill mix”OR “human resource” AND “health” OR healthcare OR maternal OR mother OR child OR newborn OR “neonat*”)]. Our priority was to select existing systematic reviews which fully or partly address apriori defined facility level interventions for improving quality of care for MNH. We excluded reviews pertaining to social support for drug abuse and chronic illneses. Reviews reporting impacts of shifting duty on physiological and biochemical indicators were also excluded as these were not included in the scope of our review. Search was conducted in the Cochrane library and Pubmed and reviews that met the inclusion criteria were selected and double data abstracted on a standardized abstraction sheet. Quality assessment of the included reviews was done using Assessment of Multiple Systematic Reviews (AMSTAR) criteria [33] as detailed in the paper 1 of this series [32]. Any disagreements between the primary abstractors were resolved by the third author. For the pre-identified interventions, which did not specifically report MNH outcomes, we have reported the impacts on other health outcomes as reported by the review authors. Estimates are reported as relative risks (RR), risk ratios (RR), risk differences (RD) or mean differences (MD) with 95 % confidence intervals (CI) where available. For detailed methodology please refer to paper 1 of the series [32].

## Findings

**Figure 1:**
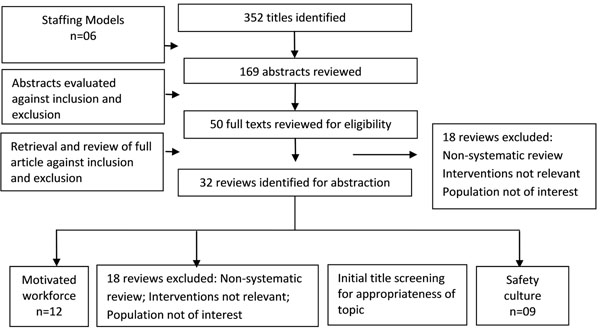
Search flow diagram

We identified 352 potentially relevant review titles and included 32 eligible reviews after further evaluation of the abstracts and full texts; 12 reviews on various aspects of well performing and motivated workforce, 5 on social support, 9 on interventions to promote safety culture and 6 reviews on staffing models (Figure 1). The overall quality of the reviews ranged from 2 to 10 with a median of 9.5 on the AMSTAR criteria.

### Well performing and motivated workforce

The quality of the 12 included reviews varied from 4 to 10 with a median of 10 on AMSTAR criteria. A range of interventions from provision of support to cope up with job stressors to exit interviews at the time of the departure from the organization were included. None of the reviews reported outcomes specific to MNH. Meta-analysis was done in only two of the reviews due to the generic nature of intervention and wide range of reported outcomes. The most commonly reported outcomes included job satisfaction, work stress, and performance. The characteristics and findings of the included reviews are presented in Table 3.

**Table 3:** Characteristics of the review included for well performing and motivated workforce

Reviews (n=12)	Description of included interventions	Type of Studies included (no)	Targeted health care providers	Outcome reported		Pooled data (Y/N)	Results
						
				Other outcomes	MNCH specific outcomes		
**Baker 2010 **[36]	Strategies to improve professional practice that are planned taking account of prospectively identified barriers to change.	26 trials (12 meta-analyzed)	Healthcare professionals responsible for patient care in HIC	Desired professional practice		Yes	1.52 (1.27- 1.82)

**Blanca-Gutierrez 2012 **[35]	Implementation of any intervention to reduce absenteeism among hospital nursing staff.	RCT: 11 observational trials: 4	Nursing staff	Nurses working full-time versus other working time		No	3.2 days on average absenteeism in nurses full-time versus 2.5 working in time partial
				Cognitive behavioral therapy			The intervention group had an average of 2.29 absences hours against 14 hours in the group control
				Flexibility of shifts (From 4 hour shifts duration up to 12 hours			41% reduction absenteeism
				Rewards			Decreased 24.97% of total days of absenteeism

**Flint 2011 **[39]	Any form of exit interview undertaken at the voluntary cessation of employment or at a prescribed time following departure from the organization was eligible. These could be a face to face exit interview, a telephone exit interview, a self-completed exit interview survey, electronic exit interview survey and mailed exit interview survey.	No trials included	Healthcare professionals	Turnover rate		No	No studies identified for inclusion

**Flodgren 2012 **[58]	An organizational infrastructure was defined as the underlying foundation or basic framework through which clinical care is delivered and supported.	ITS: 01	Healthcare organizations comprising nurses, midwives and health visitors in hospital and community settings in HIC	Risk of developing healthcare-acquired pressure ulcers (HAPUs).		No	0.7% (1.7-3.3)

**Kiwanuka 2011 **[40]	Dual practice was defined as the holding of more than one job by a health professional. Approaches identified and considered to manage dual practice were complete prohibition. Restrictions on private sector earnings, Providing incentives for exclusive public service, Raising health worker salaries, allowing private practice in public facilities, self-regulation, regulation of private sector.	None included	All health professionals in LMIC	Increased working hours, reduced waiting hours, absenteeism, reduced sick leaves		No	No studies identified for inclusion

**Parmelli 2011 **[15]	Strategy intended to change organizational culture in order to improve healthcare performance	None included	Any type of healthcare organization	Professional performance, patient outcomes		No	No studies identified for inclusion

**Pearson 2007 **[59]	Types of interventions included any strategy that had a cultural competence component, which influenced the work environment, and/or patient and nursing staff in the environment.	Descriptive:02 Qualitative:04 Discursive: 13	Staff, patients, and systems or policies that were involved or affected by concepts of cultural competence in the nursing workforce in a healthcare environment	Nursing staff outcomes, patient outcomes, organizational outcomes and systems level outcomes.		No	Appropriate and competent linguistic services, and intercultural staff training and education would contribute to the development of a culturally competent workforce.

**Peñaloza 2011 **[60]	The complex combination of factors that drives the migration flow of health professionals contributes to the complexity of the strategies to manage this flow.	ITS: 01	Any group of health professionals who are nationals of a LMIC and whose graduate training was in a LMIC.	Yearly number of Philippine nurses migrating to the USA		No	+807.6 nurses, (95% CI 480.9 to 1134.3)

**Rowe 2005 (Overview) **[61]	An essential first step towards improving performance understands the factors that influence it. Such factors fall into two categories: interventions (e.g., training) and non-intervention determinants (e.g., patient’s age).	Overview	All health workers in LMIC			No	Simple dissemination of written guidelines is often ineffective. Supervision and audit with feedback is effective. Multifaceted interventions might be more effective than single interventions

**Socha 2011 **[62]	Review of the literature on the consequences of dual practice for the physician labor supply; the quality of the public health care; the costs of the public health care provision. Section 5 discusses regulatory responses	Overview				No	Narrative

**Tanj -Dijkstra 2011 **[38]	Physical environmental stimuli are part of the (shared) healthcare environment and can be classified as ambient, architectural or interior design features that influence healthcare personnel through mediation by psychological processes.	CBA: 01	Both medical and paramedical personnel whoare directly involved in treatment and care of patients in healthcare settings.	Change in mood			Intervention group: 4.3 lower
				Satisfaction with physical environment			Not estimable
				Change in unscheduled absenteeism			Intervention group: 3.2 lower

**Van Wyk 2010 **[34]	We included any intervention intended to improve health workers’ ability to cope or manage job stress. These include: (a) formal and informal staff-support groups; (b) training or education in coping skills (or stress management) and communication; (c) management interventions, e.g. multidisciplinary meetings, feedback sessions, etc.	RCT’s: 10	Professional health workers and health teams working in primary, secondary, tertiary, community, residential and referral care settings.	**Job stress:**		Yes	**Job stress:**
				Assertiveness training vs. in-service training			-6.10 (-8.39- - 3.81)
				Stress management vs. no intervention			-0.06 (-0.44 – 0.32)
				Mindfulness training vs. no intervention			3.44 (-4.10- 10.98)
				Management intervention vs. no intervention			0.66 (-1.24 – 2.44)
				**Burnout (emotional exhaustion)**			**Burnout (emotional exhaustion)**
				Stress management vs. no intervention			-6.00 (-8.16- -3.84)
				**Job satisfaction:**			**Job satisfaction:**
				Mindfulness training vs. no intervention			1.48 (-4.81 – 7.77)
				Stress management vs. no intervention			-0.13 (-0.53 – 0.27)
				Management intervention vs. no intervention			-0.63 (-1.23- -0.03)
				**Absence:**			**Absence:**
				Management intervention vs. no intervention			20.35 (-10.65- 51.35)

Stress management trainings and management interventions for healthcare workforce involving multidisciplinary meetings and feedback sessions have reported to significantly reduce job stress (MD: -6.00, 95% CI: -8.16, -3.84) and improve job satisfaction (MD: -0.63, 95% CI: -1.23, -0.03) with no impact on absenteeism [34]. Among nursing staff reward incentives and flexible schedules reported 41% and 23% reductions in absenteeism respectively [35]. Desired professional practice such as prescribing, and adherence to recommended guidelines improved significantly (RR: 1.52, 95% CI: 1.27, 1.82) with the interventions tailored to address identified barriers to change [36]. Appropriate and competent linguistic services, and intercultural staff training and education were identified as the key components for a culturally competent workforce [37]. There was limited evidence for the impact of physical healthcare environment involving ambience, architectural or interior design features however it has reported improved staff mood and reduced unscheduled absenteeism [38]. Reviews evaluating the impact of exit interviews [39], strategies for change in organizational culture [15] and managing dual practice among healthcare workers [40] did not find any study for inclusion. Generalizability of these findings is limited to high income countries (HIC) only.

### Interpersonal care / social support

We included five reviews, with a median quality score of 10 on AMSTAR criteria. Included reviews focused on various social support strategies including support during pregnancy and special circumstance like labor, perinatal death and breast feeding. All the reviews reported MNH outcomes including birth outcomes and breast feeding duration. Meta-analysis was conducted in three reviews. Generalizability of these findings is mostly limited to HIC, as there was very limited data from low- middle-income countries (LMIC). The characteristics and finding of the included reviews are presented in Table 4.

**Table 4:** Characteristics of the review included for interpersonal care and social support

Reviews (n=5)	Description of included interventions	Type of studies included (no)	Targeted health care providers	Outcome reported		Pooled data (Y/N)	Results
						
				Outcomes	MNCH specific outcomes		
**Flenady 2008 **[43]	Any intervention provided by professional or non-professional individuals or groups aimed at improving psychological wellbeing after perinatal death.	No trials included	Professional or non-professional			N/A	No studies identified for inclusion

**Hodnett 2010 **[41]	Standardized or individualized programs of additional social support, provided in either home visits, during regular antenatal clinic visits, and/or by telephone on several occasions during pregnancy.	RCT’s: 17	Pregnant women at risk of having preterm or growth restricted babies, or both in developed countries		Antenatal hospital admission	Yes	0.79 (0.68-0.92)
					Caesarean birth		0.87 (0.78-0.97)
					Preterm birth		0.92 (0.83-1.01)
					Perinatal mortality		0.96 (0.74-1.26)

**Hodnett 2011 **[23]	Labour support by either a familiar or unfamiliar person (with or without healthcare professional qualifications).	Trials: 21	Healthcare professional (nurse, midwife) or training as a doula or childbirth educator, or be a family member, spouse/partner, friend or stranger with little or no special training in labour support in developed countries		Spontaneous vaginal birth	Yes	1.08 (1.04-1.12)
					Intrapartum analgesia		0.90 (0.84-0.97)
					Dissatisfaction		0.69 (0.59-0.79)
					Labour duration		-0.58 (-0.86 to -0.30)
					Caesarean		0.79 (0.67-0.92)
					Instrumental vaginal birth		0.90 (0.84-0.96)
					Regional analgesia		0.93 (0.88-0.99)
					Baby with a low 5-minute Apgar score		0.70 (0.50-0.96)

**Logsdon 2004 **[63]	Paraprofessional (individuals who have received specialized training in order to meet the needs of a patient population or to implement a research or project intervention) support to pregnant and parenting women.	Total : 8 studies RCT: 3 Pre-post:1 Reterospective:3 Descriptive: 1	Paraprofessionals in developed countries		Incidence of premature birth and low birth weight and small for gestational age infants, use of healthcare services, school retention in mothers and repeat pregnancies, child abuse, discipline, and maternal-infant interaction	No	Narrative

**Renfrew 2012 **[42]	‘Support’ interventions include elements such as reassurance, praise, information, and the opportunity to discuss and to respond to the mother’s questions, and it could also include staff training to improve the supportive care given to women during breast feeding.	RCT’s / Quasi: 52	Health professionals or lay people, trained or untrained, in hospital and community settings. Mostly in HIC		Stopping ‘any breastfeeding’ before 6 months	Yes	0.91 (0.88-0.96)

Standardized or individualized programs of additional social support throughout pregnancy were found to decrease the risk of antenatal hospital admission (RR: 0.79, 95% CI: 0.68, 0.92) and cesarean birth (RR: 0.87, 95% CI: 0.78, 0.97) although it did not show any impact on preterm birth, low birth weight (LBW) or perinatal mortality [41]. Support during labor was found to significantly increase spontaneous vaginal birth (RR: 1.08, 95% CI: 1.04, 1.12), reduce intra-partum analgesia (RR: 0.90; 95% CI: 0.84, 0.97), dissatisfaction (RR: 0.69; 95% CI: 0.59, 0.79), labour duration (MD: -0.58; 95% CI:-0.86, -0.30), cesarean delivery (RR: 0.79; 95% CI: 0.67, 0.92), instrumental vaginal birth (RR: 0.90; 95% CI: 0.84, 0.96), regional analgesia (RR: 0.93, 95% CI: 0.88, 0.99) and baby with a low 5-minute Apgar score (RR: 0.70, 95% CI: 0.50, 0.96)[23]. Breastfeeding support interventions including reassurance, praise, information, and staff training to improve the supportive care has shown to increase the duration and exclusivity of breastfeeding (RR for stopping any breast feeding before 6 months 0.91; 95% CI: 0.88, 0.96). These interventions are reported to be more effective in settings with high initiation rates; hence strategies to increase the uptake of breastfeeding should be in place [42]. A review evaluating the impact of supportive interventions for mothers, fathers or families after perinatal death did not find any study for inclusion [43].

### Safety culture

We included eight reviews on interventions to promote safety culture in health facility with a median AMSTAR score of 5.5; four reviews focused on administration of preventive influenza vaccination to healthcare workers and its effectiveness and uptake [44-47], one on hand hygiene promotion [48], one on the impact of interventions to reduce medication related errors [49] while two of the reviews reported the impacts of multi-component safety culture strategies and organizational interventions [50,51]. None of the reviews reported MNH specific outcomes while meta-analysis was conducted in two of the reviews. The characteristics and findings of the included reviews are presented in Table 5.

**Table 5:** Characteristics of the review included safety culture

Reviews (n=9)	Description of included interventions	Type of studies included (no)	Targeted health care providers	Outcome reported		Pooled data (Y/N)	Results
						
				Other outcomes	MNCH specific outcomes		
**Burls 2006 **[44]	Influenza vaccination	cRCT: 3 RCT: 03 Before/after studies: 05Surveys: 07	Health care workers in hospitals, nursing homes or the community in contact with high-risk individuals in HIC	Vaccination uptake		No	Range 5% - 45%
				Effectiveness			Narrative

**Gould 2011 **[48]	Hand hygiene	ITS: 02 RCT: 01 CBA: 01	Nurses, doctors and other allied health professionals (except operating theatre staff) in any hospital or community setting, (HIC)	Effectiveness		No	Multifaceted campaigns with social marketing or staff involvement appear to have an effect

**Hollmeyer 2009 **[45]	Identify self-reported reasons among HCW for vaccine acceptance or non-acceptance and to identify predictive factors that are statistically associated with influenza vaccine acceptance.	13 studies	Physicians, nurses or both and not support staff or para/non-medical personnel	Self-reported reasons		No	If HCW get immunized against influenza, they do so primarily for their own benefit and not for the benefit to their patients
				Predictive factors			

**Morello 2013 **[50]	There were a number of different safety culture strategies tested, including leadership walk rounds, structured educational programs, team-based strategies, simulation-based training programs, multi-faceted unit-based programs and multi-component organizational interventions.	cRCT: 1 Pre-Post: 7 Historically controlled studies: 13	Any study on with health care workers within a hospital, hospital department or clinical unit	Leadership walk rounds		No	2/2 studies some to moderate effect
				Multi-faceted unit-based programs			6/7 studies some to moderate effect
				Multi-component organizational strategies			1 study showed no effect
				Structured educational programs			1/2 studies some to moderate effect
				Simulation-based training programs			1/4 studies some to moderate effect
				Team based strategies			1/3 studies some to moderate effect
				Other patient safety culture strategies			1/2 studies some to moderate effect

**Nascimanto 2009 **[64]	Safety culture and patient safety	48 references	General health care environment			No	Narrative

**Ng 2011 **[46]	Influenza vaccination	RCT: 03		Mean number of working days lost		Yes	0.08 (0.19 to 0.02)
				Days with ILI symptoms			0.12 ( 0.3 to 0.06)
				RR of ILI episodes			1.14 ( 0.15 to 8.52)

**Royal 2006 **[49]	Interventions applied in primary care which aimed to reduce drug-related morbidity, hospitalization or death resulting from medication overuse or misuse.	38 studies	Pharmacist, Nurses, healthcare professionals in HIC	Hospital admission in pharmacist led intervention		Yes	0.64 (0.43- 0.96)
				Complex interventions to reduce fall in elderly			0.91 (0.68-1.21)

**Seale 2011 **[47]	Any study examining seasonal influenza vaccination (uptake, attitudes and/or programs) among Australian hospital Health care workers was included.	10 studies	Health care workers in Australia	Policies and implementation of vaccine protocols		No	16 to 77% coverage of vaccination after intervention compared to 8 to 50% coverage before intervention

**Weaver 2013 **[51]	20 studies explicitly included team training or tools to improve team communication processes, 8 explicitly included some form of executive walk rounds or interdisciplinary rounding, and 8 explicitly used comprehensive unit based safety program (CUSP).	Pre–post studies: 27 RCT: 4 Observational: 3	Any health care professionals or paraprofessionals practicing in adult or pediatric inpatient settings	**CUSP** Staff perceptions of safety culture		No	6/8 studies reported statistically significant improvements in
				Safety culture score			23/32 studies reported improvement
				Patient outcomes			6/11 studies reported improvement

Influenza vaccination among health care workers significantly reduced the mean number of working days lost (MD: 0.08,95 % CI: 0.19, 0.02) and days with influenza like illness (MD: 0.12, 95% CI: 0.3, 0.06) with non-significant impact on the risk of influenza like illness (RR: 1.14, 95 % CI: 0.15, 8.52). Programs intended to increase influenza vaccination uptake among healthcare workers reported 5%–45% increase in uptake with best case cost saving of £12/vaccine [44]. Accurate information dissemination and addressing concerns and misconceptions was identified as the key components to increase the acceptance and uptake of influenza vaccinations [46]. Pharmacist-led interventions aimed to reduce drug-related morbidity, hospitalization or death from medication overuse or misuse in healthcare facility have shown significant impact on reducing hospital admissions (RR: 0.64, 95% CI: 0.43, 0.96) although the evidence is weak and does not report impact on preventable drug related morbidity [49]. Various safety culture strategies and interventions to improve hand hygiene compliance reported insufficient evidence to draw any firm conclusion [48] with some evidence of improved perceptions and potentially reduced patient harm [50,51].

### Staffing models

We included six reviews pertaining to staffing models and skill mix with a major focus on nurses except one review focusing on the impact of collaborative care among all healthcare professionals [52]. The median AMSTAR score for the included reviews was 9. MNH specific outcomes were reported in only one review evaluating the impact of midwifery teams [53] while other reported outcomes included impact on healthcare measures like hospital stay, complications rate, mortality and re-visit rates. Meta-analysis was conducted in three reviews. The characteristics and findings of the included reviews are presented in Table 6.

**Table 6:** Characteristics of the reviews included for staffing models

Reviews	Description of included interventions	Type of studies included (no)	Targeted health care providers	Outcome reported		Pooled data (Y/N)	Results
						
				Other outcomes	MNCH specific outcomes		
**Butler 2011 **[53]	Interventions of staffing models, staffing levels, skill mix, grade mix, or qualification mix.	RCT: 08 CBA:5 CCT: 02	Hospital nursing staff and hospital patients in HIC	In-hospital mortality		Yes	0.96 (0.59-1.56)
				Length of stay			1.35 lower (1.92-0.78)
				Readmission			1.15 (0.88-1.52)
				ED within 30 days			1.14 (0.79-1.62)
				Post-discharge admission			1.33 (0.93-1.91)
				ED visit or death			1.03 (0.7 - 1.53)
				Post discharge adverse events Glycosylated hemoglobin			0.5 lower (1.9 lower – 0.9 higher)
					Medical procedures in labor		Reduced (1/1)
					Length of stay		Reduced (1/1)

**Hodgekinsons 2011 **[65]	Interventions of interest included organizational interventions (e.g. team/modular nursing, primary nursing, hierarchical nursing, care pairs or partner-in-care models) or regulatory interventions (e.g. staff patient/resident ratios).	ITS: 01 CBA: 01	Nurses and personal care attendants in HIC	• Incidence of pressure ulcers;		No	Two studies generally favour the use of primary care
				• Incidence of falls;			
				• Incidence of medication errors and adverse events;			
				• Validated quality of life measurements.			
				• Days/hours lost to sick leave;			
				• Days/hours lost to stress leave;			
				• Staff turnover rates (as a percentage of staff total);			
				• Staff burnout (as defined by the authors).			

**Kane 2007 **[55]	Nursing staffing models	7 case-control 3 case series 42 cross sectional 43 assessed temporality	Nurses in HIC	In hospital related mortality by increasing 1 RN FTE/patient day		Yes	0.92 (0.90-0.94)
				Failure to rescue by increasing 1 RN FTE/patient day			0.91 (0.89; 0.94)
				Length of stay by increasing 1 RN FTE/patient day			-0.25 (0.02)

**Kane 2007 **[54]	Various authors had used different operational definitions for the RN-to-patient ratio, including number of patients cared for by 1 RN per shift and the number of RN FTEs per patient day, 1000 patient days, or occupied bed.	17 cohort, 7 cross sectional, 4 case control,	Nurses	**Per additional full time equivalent per patient day**		Yes	**Per additional full time equivalent per patient day**
				Hospital related mortality in ICUs			0.91 (0.86-0.96
				Surgical			0.84 (0.80-0.89)
				Medical patients			0.94 (0.94-0.95)
				**An increase by 1 RN per patient day**			**An increase by 1 RN per patient day**
				Hospital acquired Pneumonia			0.70 (0.56-0.88)
				Unplanned extubation			0.49 (0.36-0.67)
				Respiratory failure			0.40 (0.27-0.59)
				Cardiac arrest			0.72 (0.62-0.84)
				Risk of failure to rescue			0.84 (0.79-0.90)
				Length of stay was shorter by 24%			0.76 (0.62-0.94)

**Thungjaroenkul 2007 **[56]	Nursing staff	17 studies: 2 prospective, 10 retrospective, 4 retrospective and prospective study, 1 Pre-post quasi-experimental design	Nurses in HIC	Patient length of stay		No	Sufficient numbers of RNs may prevent patient adverse events that cause patients to stay longer
				Hospital costs			

**Zwarenstein 2009 **[52]	A practice-based intervention introduced to a practice setting with an explicit objective of improving collaboration between two or more health and/or social care professionals.	5 RCT	Health care professionals	Health measures		No	IPC interventions can improve healthcare processes and outcomes,
				Quality of life measures			
				Complication rates			

Continuous care provided by specialized midwifery teams from early pregnancy to the postnatal period was found to reduce medical procedures in labour and resulted in a shorter length of stay without compromising maternal or perinatal safety. Under this model of care, the same midwife planned most of the care for the woman from the beginning of her pregnancy to the end of the postnatal period. However, these findings are based on a single study [53]. The addition of specialist nurses resulted in significant reduction in length of stay (1.35 lower, 95% CI: 1.92-0.78 lower) with non-significant impact on patient death rates, attendance at the emergency department, or readmission rates [53]. Self-scheduling to meet patient care demands and primary nursing (assigning one nurse for total care of a number of patients to provide comprehensive, individualized and consistent care) were reported to reduce staff turnover however, these findings are also subject to limited data availability [53]. Increasing nursing staffing in hospitals was reported to significantly reduce in-hospital mortality (RR: 0.92, 95% CI: 0.90, 0.94), failure to rescue (RR: 0.91, 95% CI: 0.89, 0.94), length of stay (mean: -0.25, SD: 0.02) and patient costs with overall better outcomes among intensive care and surgical patients [54-56]. Inter-professional rounds, meetings and audits suggested some positive impacts on healthcare processes and outcomes; however, the findings are derived from small number of studies and sample sizes involving variety of interventions and settings [50,51].

## Discussion

At the facility level, evidence suggests that social support and specialized midwifery care throughout pregnancy, labour and postnatal period have the potential to improve a range of perinatal, maternal, and labor specific indicators. However, we did not find any impact of these interventions on delivery outcomes. Among the interventions targeted at healthcare workers, stress management trainings, multidisciplinary meetings and feedback sessions can reduce work related stress and improve performance. Programs to improve influenza vaccination uptake among healthcare workers resulted in improved vaccination coverage with evidence of being cost effective as well. We found limited and inconclusive evidence on the effectiveness of exit interviews and organizational environment and cultural modifications. Most of the data from these reviews pertain to HIC hence limiting the generalizability of these findings. Notwithstanding the lack of data from LMIC, interventions like support during pregnancy and labour are expected to be effective in all settings. Moreover, such interventions would work best in resource limited settings where advanced pain relief measures are not available. Lack of evidence from LMIC may be attributable to the weak existing health system infrastructure since most of these interventions require a pre-existing healthcare infrastructure to ensure scale-up and sustainability.

Facility level inputs and reported outcomes varied widely due to diverse and complex nature of interventions involved. These interventions are by and large aimed at improving general health outcomes and health workforce performance and MNH domain can also benefit from these findings. Many of these interventions including support during pregnancy and labour, staffing and skills mixing models, increasing available workforce, improving workforce performance and safety culture promotion can be tailored and directed to improve BEmONC and CEmONC facilities and their staff performances. Proven interventions to promote staff motivation can result in enhanced support and care during pregnancy and labor and consequently result in women’s improved childbirth experience and confidence in the caregivers, which in itself is a determinant for positive pregnancy outcome [57]. Likewise, implementing standard guidelines for maternal and neonatal care can facilitate a systematic approach to evaluate and improve care provided by MNH services. It could lead to introducing routine clinical audits and enhance quality improvement processes within MNH facilities. Safety culture promotion in BEmONC and CEmONC facilities should aim at equipping them with adequate drugs, supplies and equipment for safe delivery as the quality of maternal care relies heavily on availability of functional equipment, supplies, drugs and blood for transfusion required during pregnancy and delivery.

There is a dearth of evidence on the facility level inputs from LMIC where most maternal and newborn mortality and morbidity is concentrated. There is also a need to describe individual components of the intervention and process measures in detail for reproducibility in resource limited settings. Policy makers in LMIC should focus on implementing these evidence based facility directed interventions to provide sufficient and skilled staff coupled with access to functioning equipment, drugs and supplies at the BEmONC/CEmONC facilities to provide timely and appropriate maternal and newborn care. This would consequently lead to reduced maternal and newborn mortality attributable to delayed treatment of obstetric complications.

Future studies should evaluate the effectiveness of structural and cultural changes, educational interventions, grade mix interventions, and staffing levels on workforce performance and patient outcomes. Determinants of healthcare worker performance, sustainability and cost-effectiveness should be evaluated using rigorous study designs. Further evidences are now needed to evaluate the best possible combination of strategies tailored to the need of the area of implementation.

## Abbreviations

AMSTAR: Assessment of Multiple Systematic Reviews; BEmONC: Basic Emergency Obstetric and Newborn Care; CEmONC: Comprehensive Emergency Obstetric and Newborn Care; CI: Confidence Interval; LMIC: Low- and Middle- Income Countries; MD: Mean Difference; MNH: Maternal Newborn Health; RD: Risk Difference; RR: Relative Risk

## Competing interests

We do not have any financial or non-financial competing interests for this review.

## Author contributions

All authors contributed to the process and writing of the manuscript.

## Peer review

Peer review reports are included in Additional file 1.

## Supplementary Material

Additional File 1 Click here for file

## Declarations

This article has been published as part of *Reproductive Health* Volume 11 Supplement 2, 2014: Quality of Care in Maternal and Child Health. The full contents of the supplement are available online at http://www.reproductive-health-journal.com/supplements/11/S2.
