# Supplementary material for: Evidence from facility level inputs to improve quality of care for maternal and newborn health: interventions and findings
Source: Reprod Health. 2014 Sep 4;11(Suppl 2):S4. doi: 10.1186/1742-4755-11-S2-S4 (PMC4160922; doi:10.1186/1742-4755-11-S2-S4)
Supplement: Additional File 1 [file 1742-4755-11-S2-S4-S1.pdf]

Referee's comments to the authors– this sheet WILL be seen by the author(s) and published alongside the article

Article ref no.	
Title	Evidence from community level inputs to improve quality maternal and newborn health: Interventions and findings
Author(s)	Zohra S lassi; Jai K. Das; Rehana A Salam & Zulfiqar A Bhutta
Referee's name	J. Shea

When assessing the work, please consider the following points, where applicable:

[USE THE APPROPRIATE QUESTIONS FOR THE ARTICLE TYPE TO BE REVIEWED – SEE Reviewer Guidelines above]

1. Is the question posed by the authors new and well defined?
2. Are the methods appropriate and well described, and are sufficient details provided to replicate the work?
3. Are the data sound and well controlled?
4. Does the manuscript adhere to the relevant standards for reporting and data deposition?
5. Are the discussion and conclusions well balanced and adequately supported by the data?
6. Do the title and abstract accurately convey what has been found?
7. Is the writing acceptable?

Please make your report as constructive and detailed as possible in your comments so that authors have the opportunity to overcome any serious deficiencies that you find and please also divide your comments into the following categories:

- Major Compulsory Revisions (which the author must respond to before a decision on publication can be reached)
- Minor Essential Revisions (such as missing labels on figures, or the wrong use of a term, which the author can be trusted to correct)
- **Discretionary Revisions (which are recommendations for improvement but which the author can choose to ignore)**

Where possible please supply references to substantiate your comments.

When referring to the manuscript please provide specific page and paragraph citations where appropriate.

Similar to the other papers in this review series, facility level procedures and processes are an integral component of the quality of healthcare; hence the paper is important and relevant.

Abstract: The entire Background is one sentence. The paragraph should be revised and restructured. The question/objective is not articulated.

Background: The 2nd paragraph clearly explains the focus of the paper.

Facility Level characteristics: "competency" is singular.

- "cope up" is incorrect (p.3).

Well performing and motivated workforce: Nice job in this section. Review and revise grammatically incorrect sentences.

Interpersonal care and social support: Nice job
Safety culture: Concise and clear with some minor language errors.
Staffing models: Concise and clear.

Search Strategy: The preliminary search strategy included all available systematic reviews on district level interventions as presented in the conceptual framework (presented in paper1). It seems that the initial search included a number of related but not relevant papers and these were excluded. The search strategy is generally well described, but I would advise that the authors be more specific about what keywords and what combination (strings) they searched for. It is important to note that Cochrane Systematic Reviews include only Randomised Controlled Trials (RCTs) and do not include a wide variety of other research designs, like mixed method studies. Social science and policy reviews may have been excluded from this review, and these are often excellent sources of quality of care research. The Pubmed search and "reviews that met the inclusion criteria" need to be described. General information about the study designs included in reviews other than Cochrane Systematic Reviews is recommended.

The diagram on page 19 is a good way of presenting the review process. I would recommend that some of the descriptions be expanded:
Titles screened – Initial title screening for appropriateness of topic
Abstracts screened – Abstracts evaluated against inclusion and exclusion criteria.
Full text screened – retrieval and review of full article against inclusion and exclusion criteria
All inclusion criteria met and no exclusion criteria

Findings: "The quality of the 12 included reviews varied from 4 to 10 with a median of 10" – (p.5) - insert in relation to the AMSTAR review criteria.

- Generally well-written.
- No findings on interventions provided for the health workforce were reported.
- Explanations of terms like "team midwifery"; "self-scheduling"; "primary nursing" and "specialist nurses" are necessary.

Discussion: The discussion is generally good, although there are some shortcomings that need to be addressed. The purpose of the Discussion is to debate the evidence and identify the limitations of the review. The Discussion of the evidence can be improved.

- The Discussion is not systematically linked to the facility Level Characteristics and Findings.
- It is advisable that the authors address the issues that are not linked to the evidence in their review as limitations of the review.

There are fewer language, syntax and grammar issues in this paper. However, there are minor errors, like missing commas, that can be improved.

Referee's comments to the authors– this sheet WILL be seen by the author(s) and published alongside the article

Article ref no.	
Title	Evidence from facility level inputs to improve quality of maternal and newborn health: Interventions and Findings
Author(s)	Jai K Das[1], Rohail Kumar[1], Rehana A Salam[1], Zohra S Lassi[1], Zulfiqar A Bhutta[12]
Referee's name	Viva Combs Thorsen

When assessing the work, please consider the following points, where applicable:

[USE THE APPROPRIATE QUESTIONS FOR THE ARTICLE TYPE TO BE REVIEWED – SEE Reviewer Guidelines above]

8. Is the question posed by the authors new and well defined?
9. Are the methods appropriate and well described, and are sufficient details provided to replicate the work?
10. Are the data sound and well controlled?
11. Does the manuscript adhere to the relevant standards for reporting and data deposition?
12. Are the discussion and conclusions well balanced and adequately supported by the data?
13. Do the title and abstract accurately convey what has been found?
14. Is the writing acceptable?

Please make your report as constructive and detailed as possible in your comments so that authors have the opportunity to overcome any serious deficiencies that you find and please also divide your comments into the following categories:

- Major Compulsory Revisions (which the author must respond to before a decision on publication can be reached)
- Minor Essential Revisions (such as missing labels on figures, or the wrong use of a term, which the author can be trusted to correct)
- Discretionary Revisions (which are recommendations for improvement but which the author can choose to ignore)

Where possible please supply references to substantiate your comments.

When referring to the manuscript please provide specific page and paragraph citations where appropriate.

General comments: thank you for the opportunity to review the manuscript. It is an important topic. The authors provide a summary of evidence-based interventions at the facility level that have been shown to improve MNH care. However, before a decision about whether to accept the manuscript for publication or not several issues must be address. Please read below.

Major compulsory revisions:

- **Abstract:** What is/are the objective(s) of the study? It/They should be included. The conclusion section includes results "we found limited and inconclusive evidence for the impacts of physical environment..." It should be moved to findings. A methods section should be included. The length of the abstract (400+ words) seems a bit long. Does the length comply with the guidelines?

- **Background**: Provide a brief description (perhaps in a foot note if allowed) of the signal functions for BEmOC and CEmOC. The authors mention in the first paragraph, towards the end, neonatal mortality can be reduced to 70%, what reduction percentage is observed for maternal mortality? It should be included. Though this manuscript is part of a series, it should stand alone. There should be a brief description of the conceptual framework as it relates to facilities (and not just refer readers to paper 1). Why have the authors not organize the background section according to the elements in the conceptual framework for facilities, i.e., Organizational Capacity, Appropriate Financing, Service Infrastructure-Electronic health records/electronic communication, Human Resource Training, Well-performing and Motivated Workforce? It will help in making the manuscript flow and read easily. Panel 1 on page 4 is not referred to anywhere in the text, and it's redundant to what's already written so deleting it altogether is suggested. Life-saving commodities need to be included. Currently, there are no objectives or aim/purpose of the study/paper; the authors must explicitly state it.

- **Methods:** Though this manuscript is part of a series, it should still stand alone. The methods section, therefore, needs much more details. As is others will not be able to replicate what was done. The authors should provide information on the time period for which the search/review cover, background description of who extracted the data and how were they trained (what did the training entail); a brief description of AMSTAR entail (and what cut-off scores are), and an explanation as to why the authors are reporting on general health outcomes when the focus should be solely on MNH outcomes. Also, an appendix that includes the list of pre-identified MeSH and key search terms would help the readers judge rigor and assess bias.

- **Findings:** The authors identified 352 potentially relevant review titles then ended up with 12 eligible reviews. To promote more transparency and reduce publication bias, provide more details as to what was excluded and on the figure it would be helpful to possible group the excluded ones into categories. In the tables where the authors state "Narrative" in the results section, they should provide more details; "narrative" is insufficient. Also, where authors state "none included" for the studies, they should reword to state something to the effect that no studies met the inclusion criteria, etc. Kiwanuka 2011 (Ref 57) is an intervention protocol which is why there were no studies included. Therefore the authors must obtain and review the review itself. Otherwise exclude this reference.

- **Discussion:** The discussion tended to repeat what had already been said (with streams of anectdotal thought), instead of critically thinking about the issue and providing a deeper, more profound understanding of the issues. It lacked explanations of results that were observed (e.g. the non-significant impacts on delivery outcomes, why do the authors think that is). Findings were incoherently linked (e.g. first paragraph performance and motivation linked with influenza vaccination without elaborating). Were there any findings that were surprising/unexpected, etc? What are the implications of the? The majority of the studies that were included were conducted in developed countries. It would strengthen the paper to include more discussion around this and other ways in which LMIC could glean insights from these studies. It appears as though the authors have introduced new findings/concepts: "structural and cultural changes, educational interventions and grade mix interventions" without adequate discussion. Subheadings will help the reader (which would include, for example, limitations, future research, recommendations, and conclusion). What are the limitations and strengths of this study? A discussion about the AMSTAR scores would also be useful (what are their implications?).

- **English editing is required.**

Minor essential revisions:

- Spacing of citations within the text—sometimes there is a space before the reference number and sometimes there isn't.
- Consistency in what's included in the tables, e.g. sometimes the authors provide details about the type of studies included (in a few of them they don't); the same with the target group (i.e., whether they involve LMIC or HIC)

Discretionary revisions:

- The title: is it not a matter of improving not only the quality of maternal and newborn health but also the "care" provided? Should that be reflected in the title (both health outcomes and quality of care)?
- Consistency in terminology: facility level interventions vs. facility level characteristics vs. facility level inputs
- I don't see the relevance of including influenza vaccination uptake if the focus is in fact in LMIC where the highest burden of MN mortalities & morbidities are. The authors should seriously consider excluding references numbers 42 and 44.

Author responses

Major compulsory revisions:

- **Abstract:** What is/are the objective(s) of the study? It/They should be included. The conclusion section includes results "we found limited and inconclusive evidence for the impacts of physical environment..." It should be moved to findings. A methods section should be included. The length of the abstract (400+ words) seems a bit long. Does the length comply with the guidelines?

Thanks. We have now added objective and modified the abstract as suggested. Also reduced the length of the abstract to comply with the guidelines.

- **Background**: Provide a brief description (perhaps in a foot note if allowed) of the signal functions for BEmOC and CEmOC. The authors mention in the first paragraph, towards the end, neonatal mortality can be reduced to 70%, what reduction percentage is observed for maternal mortality? It should be included. Though this manuscript is part of a series, it should stand alone. There should be a brief description of the conceptual framework as it relates to facilities (and not just refer readers to paper 1). Why have the authors not organize the background section according to the elements in the conceptual framework for facilities, i.e., Organizational Capacity, Appropriate Financing, Service Infrastructure-Electronic health records/electronic communication, Human Resource Training, Well-performing and Motivated Workforce? It will help in making the manuscript flow and read easily. Panel 1 on page 4 is not referred to anywhere in the text, and it's redundant to what's already written so deleting it altogether is suggested. Life-saving commodities need to be included. Currently, there are no objectives or aim/purpose of the study/paper; the authors must explicitly state it.

Thanks. We have now provided a brief description of the signal functions in the background and also added a lifesaving list of interventions and commodities. We have now organized the background as suggested. We have now added a clear objective.

- **Methods:** Though this manuscript is part of a series, it should still stand alone. The methods section, therefore, needs much more details. As is others will not be able to replicate what was done. The authors should provide information on the time period for which the search/review cover, background description of who extracted the data and how were they trained (what did the training entail); a brief description of AMSTAR entail (and what cut-off scores are), and an explanation as to why the authors are reporting on general health outcomes when the focus should be solely on MNH outcomes. Also, an appendix that includes the list of pre-identified MeSH and key search terms would help the readers judge rigor and assess bias.

Thanks for pointing this out. Although the detailed methodology is described in paper 1 of this series, we have now expanded the methodology section for this paper to include search time period, pre-identified MeSH and key search terms. We have also referenced paper 1 in this section that gives a detailed methodology and quality assessment methods. It was an apriori defined criteria to include and report findings from reviews on other health outcomes, if there was no evidence reported on MNH specific outcomes.

- **Findings:** The authors identified 352 potentially relevant review titles then ended up with 12 eligible reviews. To promote more transparency and reduce publication bias, provide more details as to what was excluded and on the figure it would be helpful to possible group the excluded ones into categories. In the tables where the authors state "Narrative" in the results section, they should provide more details; "narrative" is insufficient. Also, where authors state "none included" for the studies, they should reword to state something to the effect that no studies met the inclusion criteria, etc. Kiwanuka 2011 (Ref 57) is an intervention protocol

which is why there were no studies included. Therefore the authors must obtain and review the review itself. Otherwise exclude this reference.

We have now added the reasons for exclusion in figure 1. We had now replaced "narrative" with the narrative findings of the reviews. We have now replaced "none included" with "no studies identified for inclusion". Kiwanuka 2011 was a wrong reference. Thanks for pointing this out. We have now corrected it.

- **Discussion:** The discussion tended to repeat what had already been said (with streams of anectdotal thought), instead of critically thinking about the issue and providing a deeper, more profound understanding of the issues. It lacked explanations of results that were observed (e.g. the non-significant impacts on delivery outcomes, why do the authors think that is). Findings were incoherently linked (e.g. first paragraph performance and motivation linked with influenza vaccination without elaborating). Were there any findings that were surprising/unexpected, etc? What are the implications of the? The majority of the studies that were included were conducted in developed countries. It would strengthen the paper to include more discussion around this and other ways in which LMIC could glean insights from these studies. It appears as though the authors have introduced new findings/concepts: "structural and cultural changes, educational interventions and grade mix interventions" without adequate discussion. Subheadings will help the reader (which would include, for example, limitations, future research, recommendations, and conclusion). What are the limitations and strengths of this study? A discussion about the AMSTAR scores would also be useful (what are their implications?).
  Thanks. We have now substantially modified the discussion as suggested

- **English editing is required.**

Minor essential revisions:

- Spacing of citations within the text—sometimes there is a space before the reference number and sometimes there isn't. we have now tried to correct that.
- Consistency in what's included in the tables, e.g. sometimes the authors provide details about the type of studies included (in a few of them they don't); the same with the target group (i.e., whether they involve LMIC or HIC). We have now tried to make the table consistent

Discretionary revisions:

- The title: is it not a matter of improving not only the quality of maternal and newborn health but also the "care" provided? Should that be reflected in the title (both health outcomes and quality of care)? We have slightly modified the title
- Consistency in terminology: facility level interventions vs. facility level characteristics vs. facility level inputs. Corrected
- I don't see the relevance of including influenza vaccination uptake if the focus is in fact in LMIC where the highest burden of MN mortalities & morbidities are. The authors should seriously consider excluding references numbers 42 and 44.

Paper 4. Evidence from facility level inputs to imrove quality of maternal and newborn health: Intervention and Findings

The authors have to address the following issues:

1. The fact that LMIC-countries have a relatively weak functional health care service infrastructure does not make the evidence that continuous support during labour is effective in reducing obstetric interventions cannot be generalized to LMIC-countries. This is non-sense; of course continuus support during labour would work similar to what we know from "developed countries". One of the important conclusions of the Cochrane is that continuous support is especially working in settings where there is no pain relief (which is essential what is the case in LMIC). Agreed and we have now rephrased the sentences for more clearly.
2. When the authors write on page 4: "Pregnancy, perinatal deaths, childbirth and parenting are some of the specific phenomenon that requires continuous support." I agree completely, but this is true everywhere on the globe and we don't need new studies in LMIC to show this. We just have to implement this and in the discussion (of paper 5 the authors have to address the issue: why is an effective intervention which does not cost a single penny not implemented in LMIC? Agreed. We have now added this point in our discussion.
3. On page 5 and 6 you mention: "reduce job stress (MD: -6.00, 95% CI: -8.16 - -3.84) and "improve job satisfaction (MD: -.63, 95% CI: -1.23- -0.03). Both reduce and improve give minus results, I don't follow this. For job satisfaction, low scores indicate positive feelings.
4. Again I miss the papers on audit and perception of audit on health care workers both from Malawi and Tanzania. You might not be able to find individual studies in the paper since we have conducted and overview of the systematic reviews
5. On page 8: lack of data on educational interventions: here I miss two papers I know and may be many more (I did not search): Bakker W et al. Trop Med Int Health 2011; 16: 1243-50 and Van hamersveldt KT at al. Trop med Int Health 2012; 17: 652-7. Another paper is Ouedraogo RF et al. BJOG 2009; 116: 38-44. We have checked the references that you have highlighted. They are not included in our review as these are individual studies or non-systematic reviews.

Abstract: The entire Background is one sentence. The paragraph should be revised and restructured. The question/objective is not articulated. Thanks. We have now revised the abstract as suggested and also added the research question

Background: The 2nd paragraph clearly explains the focus of the paper. Thanks

Facility Level characteristics: "competency" is singular.

- "cope up" is incorrect (p.3). corrected

Well performing and motivated workforce: Nice job in this section. Review and revise grammatically incorrect sentences. Corrected

Interpersonal care and social support: Nice job. Thanks

Safety culture: Concise and clear with some minor language errors. Thanks and corrected

Staffing models: Concise and clear. Thanks

<u>Search Strategy</u>: The preliminary search strategy included all available systematic reviews on district level interventions as presented in the conceptual framework (presented in paper1). It seems that the initial search included a number of related but not relevant papers and these were excluded. The search strategy is generally well described, but I would advise that the authors be more specific about what keywords and what combination (strings) they searched for. It is important to note that Cochrane Systematic Reviews include only Randomised Controlled Trials (RCTs) and do not include a wide variety of other research designs, like mixed method studies. Social science and policy reviews may have been excluded from this review, and these are often excellent sources of quality of care research. The Pubmed search and "reviews that met the inclusion criteria" need to be described. General information about the study designs included in reviews other than Cochrane Systematic Reviews is recommended. Thanks. We have now added key term used and also reasons for exclusion in figure 1. We have mentioned the types of study designs included in the review the characteristics of the reviews table for ready reference.

The diagram on page 19 is a good way of presenting the review process. I would recommend that some of the descriptions be expanded:

Titles screened – Initial title screening for appropriateness of topic

Abstracts screened – Abstracts evaluated against inclusion and exclusion criteria.

Full text screened – retrieval and review of full article against inclusion and exclusion criteria

All inclusion criteria met and no exclusion criteria

Modified as suggested

Findings: "The quality of the 12 included reviews varied from 4 to 10 with a median of 10" – (p.5) - insert in relation to the AMSTAR review criteria. Added

- Generally well-written. Thanks
- No findings on interventions provided for the health workforce were reported. Findings for healthcare workforce reported under "well performing and motivated workforce"
- Explanations of terms like "team midwifery"; "self-scheduling"; "primary nursing" and "specialist nurses" are necessary.Added

Discussion: The discussion is generally good, although there are some shortcomings that need to be addressed. The purpose of the Discussion is to debate the evidence and identify the limitations of the review. The Discussion of the evidence can be improved.

- The Discussion is not systematically linked to the facility Level Characteristics and Findings.
- It is advisable that the authors address the issues that are not linked to the evidence in their review as limitations of the review.

There are fewer language, syntax and grammar issues in this paper. However, there are minor errors, like missing commas, that can be improved.

We have now substantially modified the discussion as suggested
